# Conflicting evidence combination based on uncertainty measure and distance of evidence

**DOI:** 10.1186/s40064-016-2863-4

**Published:** 2016-07-29

**Authors:** Wen Jiang, Miaoyan Zhuang, Xiyun Qin, Yongchuan Tang

**Affiliations:** School of Electronics and Information, Northwestern Polytechnical University, Xi’an, 710072 Shaanxi China

**Keywords:** Dempster–Shafer evidence theory, Conflict, Information fusion, Belief entropy, Distance of evidence

## Abstract

Dempster–Shafer evidence theory is widely used in many fields of information fusion. However, the counter-intuitive results may be obtained when combining with highly conflicting evidence. To deal with such a problem, we put forward a new method based on the distance of evidence and the uncertainty measure. First, based on the distance of evidence, the evidence is divided into two parts, the credible evidence and the incredible evidence. Then, a novel belief entropy is applied to measure the information volume of the evidence. Finally, the weight of each evidence is obtained and used to modify the evidence before using the Dempster’s combination rule. Numerical examples show that the proposed method can effectively handle conflicting evidence with better convergence.

## Background

In practical applications, the information collected from the sensors is often imprecise and uncertain. How to deal with the uncertain information effectively is still an open issue. To address the uncertain information, many math tools are presented such as fuzzy sets theory (Zadeh 1965; Jiang et al. 2015), evidence theory (Dempster 1967; Shafer 1977), rough sets theory (Walczak and Massart 1999; Greco et al. 2001), Z numbers (Zadeh 2011; Kang et al. 2012; Jiang et al. 2016e) and D numbers theory (Deng et al. 2014; 2015a). Also sometimes, the methods with mixed intelligent algorithms are used for decision making or related problems (Deng et al. 2015b, c; Jiang et al. 2016b; Deng 2015c).

Dempster–Shafer evidence theory (Dempster 1967; Shafer 1977) (D–S evidence theory) was introduced by Dempster and then developed by Shafer. As an uncertainty reasoning tool, it can efficiently cope with imprecise and uncertain information without prior information, so it is widely used in many fields of information fusion (Jiang et al. 2016c; Deng 2015a). However, the counter-intuitive results may be obtained when dealing with highly conflicting evidence (Zadeh 1986; Jiang et al. 2016d). If this problem cannot be solved effectively, it will greatly limit the application of D–S evidence theory. Many approaches were proposed to resolve the problem (Lefevre et al. 2002; Murphy 2000; Smets 2007). In general, there are mainly two types of methodologies. One is to modify Dempster’s combination rule, and the other is to pre-process the data. Yager (1987) and Smets (2000), solve the problem of counter intuitive combination results through redistribution conflicting evidence. In the work of Murphy (2000), Deng et al. (2004), they pre-processed the data and also achieved ideal effect, based on the Dempster’s combination rule.

Among these two methods to solve the counter-intuitive results, according to the first type of methodology, the counter-intuitive behaviors are imputed to the combination rule. However, modifying combination rule usually destroys the good properties such as commutativity and associativity. What’s more, modifying the evidence combination rule is blamed to be unreasonable if the counter-intuitive results are caused by sensor failure. Based on the analysis above, researchers prefer to modify data model, in other words, through pre-process evidence to solve the problem of highly conflicting evidence.

As to the method of pre-processing evidence, the weight vector is difficult to determine. At present, most of the researchers are limited to basing on the relationship among evidence or the evidence itself to generate the weight, instead of considering these two aspects. Murphy’s method (Murphy 2000) is just a simple arithmetic mean, which does not take into account the relationship and difference among evidence. Deng et al.’s weighted average method (Deng et al. 2004) uses the distance of evidence to determine the weight, which makes up the shortage of Murphy’s method to some degree, but the effect of evidence itself on weight is ignored.

In this paper, a method of modifying the evidence is used to solve the combination problem of conflicting evidence. By considering the difference among evidence and the effect of evidence itself on weight, a combination method based on the distance of evidence (Jousselme et al. 2001) and a novel belief entropy (Deng 2015c) is proposed. First, the novel belief entropy is introduced to measure the uncertainty. Then, according to the distance of evidence, the evidence is divided into credible evidence and incredible evidence, and to be assigned with appropriate weight based on the distance and the uncertainty. Finally, the obtained weight is used to modify the evidence to get the weighted averaging evidence and combine it ($n - 1$) times by the Dempster’s combination rule. The experimental results show that the proposed method can effectively deal with the highly conflicting evidence.

This rest of this paper is organized as follows. The basic concepts are briefly introduced in “Preliminaries” section. In “The proposed method” section, a new combination method based on the distance of evidence and Deng entropy is presented. A numerical example is illustrated to show the efficiency of this new method in “Numerical example” section. Finally, a conclusion is presented in “Conclusion” section.

## Preliminaries

In this section, some preliminaries are briefly introduced below.

### Dempster–Shafer evidence theory (Dempster 1967; Shafer 1977)

D–S evidence theory gained increasing interest in the field of information fusion. In this subsection, D–S evidence theory is briefly introduced.

#### **Definition 1**

Let $\Theta$ be the set of N mutually exclusive and exhaustive hypotheses. This set is called the frame of discernment, and defined as $\Theta = \{\theta _1, \theta _2,\ldots ,\theta _n\}$. The concept of basic probability assignment (BPA) $m{:}\,2^\Theta \rightarrow [0, 1]$, which is defined as follows:

1 $$\begin{aligned} \left\{ \begin{array}{l} m({{\varnothing} } )=0\\ \sum\nolimits _{A \subseteq \Theta }m(A)=1 \end{array} \right. \end{aligned}$$

If $m(A)> 0$, *A* is called a focal element. The BPA reflects the degree of evidence support for the proposition of *A* in frame of discernment.

#### **Definition 2**

When multiple independent BPAs are available, the combined evidence can be obtained based on the Dempster’s combination rule as follows:

2 $$\begin{aligned} m(A)=\left\{ \begin{array}{ll} \frac{\sum \nolimits _{B\cap C=A}m_1(B)m_2(C)}{1-k}&{}\quad A \ne {\varnothing} \\ 0&{}\quad A={\varnothing} \end{array} \right. \end{aligned}$$

where $k=\sum \nolimits _{B\cap C={\varnothing} }{m_1(B)m_2(C)}$, represents the degree of conflict between $m_1$ and $m_2$. The greater the *k*, the greater the degree of conflict; the smaller the *k*, the smaller the degree of conflict. $k=0$ corresponds to the evidence do no conflict; whereas $k=1$ implies complete contradiction, i.e., none of the combining masses intersect, hence the combination between $m_1$ and $m_2$ does not exist.

### Jousselme distance (Jousselme et al. 2001)

Jousselme distance is defined with the BPAs as a particular case of vectors in a $2^{|\Theta |}$-linear space. It is an appropriate measure of the difference-or the lack of similarity-between any two BPAs. In this subsection, Jousselme distance is briefly introduced.

#### **Definition 3**

Let $m_1$ and $m_2$ be two BPAs on the same frame of discernment $\Theta$. The distance between $m_1$ and $m_2$ is:

3 $$\begin{aligned} d({m_1},{m_2})=\sqrt{\frac{1}{2}(\vec {m_1}-\vec {m_2})^T \underline{\underline{D}}(\vec {m_1}-\vec {m_2})}, \end{aligned}$$

where $\underline{\underline{D}}$ is an $2^{|\Theta |}\times 2^{|\Theta |}$ matrix whose element are

4 $$\begin{aligned} D(A,B)=\frac{|A\cap B|}{|A\cup B|}\ \ \ \ \ A,B\in 2^\Theta \end{aligned}$$

### Ambiguity measure (Jousselme et al. 2006)

In evidence theory, the common uncertainty includes: nonspecificity measure (NS) (Didier and Prade 1985), aggregated uncertainty measure (AU) (Harmanec and Klir 1994) and ambiguity measure (AM) (Jousselme et al. 2006; Yang and Han 2016). And AM is widely used in uncertainty measure, which is defined as follows.

#### **Definition 4**

Suppose that $\Theta$ be a frame of discernment, *m* is a BPA, ambiguity measure (AM) is defined as follows:

5 $$\begin{aligned} AM(m)=-\sum _{\theta \in \Theta } BetP_m(\theta )\log _2(BetP_m(\theta )) \end{aligned}$$

where

6 $$\begin{aligned} BetP_m(\theta )=\frac{\sum \nolimits _{B\in \Theta } m(B)}{|B|} \end{aligned}$$

is the Pignistic probability proposed by Smets and Kennes (1994).

### Belief entropy (Deng 2015c)

Recently, a novel belief entropy, named as Deng entropy, is applied to measure the information volume of the evidence. This entropy is the generalization of Shannon entropy (Shannon 1948). It is an efficient way to measure uncertainty, not only under the situation where the uncertainty is represented by a probability distribution, but also under the situation where the uncertainty is represented by BPAs. When the uncertainty is expressed in the form of a probability distribution, the entropy definitely degenerates to Shannon entropy. The related concepts are given below.

#### **Definition 5**

Deng entropy is defined as follows:

7 $$\begin{aligned} E_d=-\sum _i m(B_i)\log \frac{m(B_i)}{2^{|B_i|}-1} \end{aligned}$$

where $B_i$ is a proposition in BPAs *m*, and $|B_i|$ is the cardinality of $B_i$.

Specially, this entropy can definitely degenerate to the Shannon entropy if the belief is only assigned to single element. Namely,

8 $$\begin{aligned} {E_d} = - \sum \limits _i m ({C_i})\log \frac{{m({C_i})}}{{{2^{\left| {{C_i}} \right| }} - 1}} = - \sum \limits _i m ({C_i})\log m({C_i}) \end{aligned}$$

In addition, the belief entropy has been used in Jiang et al. (2016a) and Yuan et al. (2016). For more detailed information, please refer to Deng (2015c) and Fei et al. (2015).

## The proposed method

Suppose that there are *n* evidence $m_i\;i=1,\ldots , n,$ the pre-processing of the evidence can be illustrated as:

9 $$\begin{aligned} \left\{ \begin{array}{ll} m=\sum \nolimits _{i=1}^n {{w_i} {m_i}}\\ \sum \nolimits _{i=1}^n {w_i}=1 \end{array} \right. \end{aligned}$$

where $w_i$ is the corresponding weight of evidence $m_i$.

In Eq. (9), each ${w_i} {m_i}$ can be considered as the discounted $m_i$, and *m* denotes the weighted averaging evidence of the original *n* evidence. The *n* evidence are weighted average according to all the available focal elements, respectively. But how to get the appropriate weight? We argue that both the distance of evidence and the uncertainty should be used to generate the appropriate weight of evidence.

The uncertainty (Jousselme et al. 2006) of evidence can reflect the clarity of the evidence: the smaller the uncertainty of evidence, the clearer the evidence, and the higher the credibility. Based on the speciality of uncertainty, we take the uncertainty as one of the factors to determine the credibility of the evidence, then it is used to determine the weight.

In D–S evidence theory, AM is widely used in uncertainty measure. However, to a certain degree, AM may be lack of information in the Pignistic probability conversion process. Compared to the AM, the calculation of the novel belief entropy (Deng 2015c) is more convenient, and can better measure the uncertainty of the evidence.

A example is given to show the effectiveness of this belief entropy below.

### *Example 1*

Assume there are two BPAs in the frame of discernment $\Theta = \{a, b, c\}$, and the BPAs are listed as follows:

$$\begin{aligned} {m_1}{:}\,{m_1}(a)&= {m_1}(b) = {m_1}(c) = 1/3;\\ {m_2}{:}\,{m_2}(a)&= {m_2}(b) = {m_2}(c) =0.05,\quad {m_2}(a,b,c) = 0.85. \end{aligned}$$

The uncertainty of each method is calculated respectively by Eqs. (5) and (7):

$$\begin{aligned} {\mathrm{AM}}{:}\,AM({m_1}) = 1.5850,\;AM({m_2}) = 1.5850; \end{aligned}$$

The novel belief entropy: ${E_d}({m_1}) = 1.5850,\;{E_d}({m_2}) = 3.2338.$

From an intuitive point of view, $m_1$ has more higher certainty than $m_2$, namely, the uncertainty of $m_2$ is greater than $m_1$. Whereas the AM of these two BPAs is the same, the result of this belief entropy is consistent with intuition.

In order to get the appropriate weight, the novel entropy is used to measure the uncertainty of the evidence, and a new combination method is proposed based on the entropy and the distance of evidence. Meanwhile, some definitions are presented below.

Suppose there are *n* mutually exclusive evidence.

1. If the Jousselme distance between one evidence and other evidence is small, namely, the evidence is supported by other evidence, then the evidence is considered as credible evidence;
2. If the Jousselme distance between one evidence and other evidence is great, namely, the evidence is not supported by other evidence, then the evidence is considered as incredible evidence.

For a credible evidence, the smaller the entropy, the smaller the uncertainty, then the clearer the evidence and it is favorable to making decision. So, the greater weight should be assigned to the evidence. On the contrary, for a incredible evidence, the smaller the entropy, the smaller the uncertainty, then the clearer the evidence. However, since there is a great conflict between the incredible evidence and other evidence, and in order to weaken its negative effects, the smaller weight should be assigned to the evidence. Based on this idea, a reward function and a penalty function are defined to generate weight.

### **Definition 6**

A reward function is defined as:

10 $$\begin{aligned} {\alpha _i} = \exp \left( - \overline{{E_d}}(m_i)\right) ,\quad i=1,2,\ldots ,n \end{aligned}$$

where $\overline{{E_d}}(m_i)$ is the normalized belief entropy, namely, $0\le \overline{{E_d}}(m_i)\le 1$.

This presented reward function meets the following properties:

### **Property 1**

*The reward function is always positive, i.e.,*$\alpha _i>0$.

### *Proof*

According to the mathematic properties of the exp-function, we can get that $\alpha _i>0$.

### **Property 2**

*The reward function is a monotone decreasing function*.

### *Proof*

According to the mathematic properties of the exp-function, we can get that this reward function is a monotone decreasing function. Thus, it can achieve that the smaller the entropy, the greater the weight. In the method, the reward function is used to generate the weight for the credible evidence.

### **Definition 7**

A penalty function is defined as:

11 $$\begin{aligned} {\alpha _i} = \exp \left[ { - \left( {\overline{E_d^{max}} + 1 - \overline{E_d}}(m_i) \right) } \right] ,\quad i=1,2,\ldots ,n \end{aligned}$$

where $\overline{E_d ^{max}}$ is the maximum normalized belief entropy. $\overline{{E_d}}(m_i)$ is the normalized belief entropy, namely, $0\le \overline{{E_d}}(m_i)\le 1$.

This presented penalty function meets the following properties:

### **Property 3**

*The penalty function is always positive, i.e.*, $\alpha _i>0$.

### *Proof*

According to the mathematic properties of the exp-function, we can get that $\alpha _i>0$.

### **Property 4**

*The penalty function is a monotone increasing function*.

### *Proof*

Arbitrary take variables $\overline{{E_d}}(m_1)$ and $\overline{{E_d}}(m_2)$, and suppose $\overline{{E_d}}(m_2)>\overline{{E_d}}(m_1)$, that is $\overline{{E_d}}(m_2)-\overline{{E_d}}(m_1)>0$, then

$$\begin{aligned} {\alpha _2}-{\alpha _1}= & {} \exp \left[ -\left( \overline{E_d^{max}}+1 - \overline{{E_d}}(m_2)\right) \right] -\exp \left[ -\left( \overline{E_d^{max}}+1 - \overline{{E_d}}(m_1)\right) \right] \\= & {} \exp \left[ \left( \overline{{E_d}}(m_2)- \overline{{E_d}}(m_1)\right) \right] \\ \end{aligned}$$

Since

$$\begin{aligned} \overline{{E_d}}(m_2)-\overline{{E_d}}(m_1)>0. \end{aligned}$$

Based on the mathematic properties of the exp-function, we can get that:

$$\begin{aligned} \exp \left[ (\overline{{E_d}}(m_2)- \overline{{E_d}}(m_1))\right] >\exp (0)=1. \end{aligned}$$

Hence

$$\begin{aligned} {\alpha _2}-{\alpha _1}>0,\quad i.e., {\alpha _2}>{\alpha _1}. \end{aligned}$$

Namely, the penalty function is a monotone increasing function. So it can achieve that the smaller the entropy, the smaller the weight. In the method, the penalty function is used to generate the weight for the incredible evidence.

Assume there are *n* evidence $m_i\; i=1,2, \ldots , n$, the building steps of weight are given below:

**Step 1** Obtain the Jousselme distances $d_{ij}\;i,j=1,2, \ldots , n$ of every two evidence $m_i$ and $m_j$ by Eq. (3). The distance matrix *DM* is given as follows:

12 $$\begin{aligned} DM = [{d_{ij}}]=\left[ {\begin{array}{cccc} 0&{}\quad {{d_{12}}}&{}\quad \cdots &{}\quad {{d_{1n}}}\\ {{d_{21}}}&{}\quad 0&{}\quad \cdots &{}\quad {{d_{2n}}}\\ \vdots &{}\quad \vdots &{}\quad \vdots &{}\quad \vdots \\ {{d_{n1}}}&{}\quad {{d_{n2}}}&{}\quad \cdots &{}\quad 0 \end{array}} \right] \end{aligned}$$

**Step 2** Calculate the average evidence distance $\overline{d_i}$ of the evidence $m_i$,

13 $$\begin{aligned} \overline{d}_i=\frac{\sum \nolimits _{j=1,j\ne i}^{n}d_{ij}}{n-1},\quad i,j=1,2,\ldots ,n \end{aligned}$$

**Step 3** Calculate the global evidence distance *d*.

14 $$\begin{aligned} d = \frac{{\sum \nolimits _{i = 1}^n {\overline{{d_i}} } }}{n},\quad i=1,2,\ldots ,n \end{aligned}$$

**Step 4** The evidence set is divided into two parts: the credible evidence and the incredible evidence.

If $\overline{d_i} \le d$, then $m_i$ is a credible evidence;

If $\overline{d_i} > d$, then $m_i$ is a incredible evidence.

**Step 5** Calculate the belief entropy ${E_d}(m_i),\;i = 1,2, \ldots , n$, and normalize it as follows.

15 $$\begin{aligned} \overline{{E_d}}(m_i) = \frac{{{E_d}(m_i)}}{{\sum \nolimits _{i = 1}^n {{E_d}(m_i)} }},\quad i=1,2,\ldots ,n \end{aligned}$$

**Step 6** Obtain the corresponding initial weight $\alpha _i,\;i = 1,2, \ldots , n$ as follows:

1. For the credible evidence, the reward function is used to generate the initial weight $\alpha _i$ according to Eq. (10);
2. For the incredible evidence, the penalty function is used to generate the initial weight $\alpha _i$ according to Eq. (11).

**Step 7** The final weight $w_i$ of evidence $m_i$ are normalized as follows:

16 $$\begin{aligned} {w_i} = \frac{{{\alpha _i}}}{{\sum \nolimits _{i = 1}^n {{\alpha _i}} }},\quad i = 1,2 \ldots ,n \end{aligned}$$

According to Eq. (9), we can get the weighted averaging evidence by the weighted average method of multi-source evidence after obtaining the weight of each evidence. Finally, the new evidence is combined for ($n-1$) times by Dempster’s combination rule and the fusion result can be obtained.

It should be pointed out that, if there are only two BPAs, the Jousselme distance loses efficacy, the building steps of weight are given as follows:

**Step 1** Calculate the belief entropy ${E_d}(m_i),\;i = 1,2$, and normalize it by Eq. (15).

**Step 2** The weight $\alpha _{i1}$ is obtained according to the reward function.

17 $$\begin{aligned} {\alpha _{i1}} = \exp \left( -\overline{{E_d}}(m_i)\right) ,\quad i = 1,2 \end{aligned}$$

**Step 3** The weight $\alpha _{i2}$ is obtained according to the penalty function.

18 $$\begin{aligned} {\alpha _{i2}} = \exp \left[ { - \left( {\overline{E_d^{max}} + 1 - \overline{E_d}}(m_i) \right) }\right] ,\quad i = 1,2 \end{aligned}$$

where $\overline{E_d ^{max}}$ is the maximum normalized belief entropy.

**Step 4** A average weight $\alpha _i$ is given as follows:

19 $$\begin{aligned} \alpha _i=\frac{1}{2}({\alpha _{i1}}+\alpha _{i2}),\quad i = 1,2 \end{aligned}$$

**Step 5** The final weight $w_i,\;i = 1,2$ of evidence $m_i,\;i = 1,2$ are normalized by Eq. (16).

## Numerical example

In this section, a simple example is given to show the efficiency of the new method.

### *Example 2*

In a multisensor-based target recognition system, there are totally three types of targets: $\Theta =\{A,B,C\}$. Suppose there are five sensors, and five acquired BPAs are listed as follows:

$$\begin{aligned} m1{:}\,m_1(A)&= {} 0.41,\ m_1(B)=0.29,\ m_1(C)=0.30;\\ m2{:}\,m_2(A)&= 0.00,\ m_2(B)=0.90,\ m_2(C)=0.10;\\ m3{:}\,m_3(A)&= 0.58,\ m_3(B)=0.07,\ m_3(A,C)=0.35;\\ m4{:}\,m_4(A)&= 0.55,\ m_4(B)=0.10,\ m_4(A,C)=0.35;\\ m5{:}\,m_5(A)&= 0.60,\ m_5(B)=0.10,\ m_5(A,C)=0.30.\\ \end{aligned}$$

Firstly, according to Eq. (3), the Jousselme distance of every two evidence can be calculated based on the initial evidence, and the distance matrix *DM* can be obtained as follows:

$$\begin{aligned} DM=\left[ \begin{array}{cccccc} 0&{}\quad 0.5386 &{}\quad 0.2892 &{}\quad 0.2699 &{}\quad 0.2848\\ 0.5386 &{}\quad 0 &{}\quad 0.7195 &{}\quad 0.6901 &{}\quad 0.7106\\ 0.2892 &{}\quad 0.7195 &{}\quad 0 &{}\quad 0.0300 &{}\quad 0.0255\\ 0.2699 &{}\quad 0.6901 &{}\quad 0.0300 &{}\quad 0 &{}\quad 0.0354\\ 0.2848 &{}\quad 0.7106 &{}\quad 0.0255 &{}\quad 0.0354 &{}\quad 0\\ \end{array} \right] \end{aligned}$$

Then, adopt Eq. (13) to calculate the average evidence distance $\overline{d_i}, i = 1,2,3,4,5$, which is given below:

$$\begin{aligned} \overline{d}_1= 0.3456,\; \; \;\; \;\overline{d}_2=0.6647,\; \; \;\; \; \overline{d}_3=0.2661,\; \; \;\; \;\overline{d}_4=0.2564,\; \; \; \;\;\overline{d}_5=0.2641. \end{aligned}$$

Secondly, the global evidence distance *d* can be obtained by Eq. (14):

$$\begin{aligned} d=0.3594 \end{aligned}$$

Thirdly, from the above, we can know that $m_1, m_3, m_4, m_5$ are the credible evidence and $m_2$ is the incredible evidence.

Additionally, the belief entropy ${E_d}(m_i), i = 1,2,3,4,5$ is calculated according to Eq. (7):

$$\begin{aligned} {{E_d}}(m_1)&= 1.5664,\;\;\;\;\;{{E_d}}(m_2) = 0.4690,\;\;\;\;\;{{E_d}}(m_3) = 1.8092,\\ {{E_d}}(m_4)&= 1.8914,\;\;\;\;\;{{E_d}}(m_5) = 1.7710. \end{aligned}$$

The results after normalization of this entropy $\overline{{E_d}}(m_i), i = 1,2,3,4,5$ according to Eq. (15) are as follows:

$$\begin{aligned} \overline{{E_d}}(m_1)&= 0.2087,\;\;\;\;\;\overline{{E_d}}(m_2) = 0.0625,\;\;\;\;\;\overline{{E_d}}(m_3) = 0.2410,\\ \overline{{E_d}}(m_4)&= 0.2520,\;\;\;\;\;\overline{{E_d}}(m_5) = 0.2359. \end{aligned}$$

Then, the weight $\alpha _i, i = 1,2,3,4,5$ of the credible evidence and the incredible evidence can be obtained based on Eqs. (10) and (11), respectively.

$$\begin{aligned} {\alpha _1}&= \exp ( - 0.2087)=0.8117;\\ {\alpha _2}&= \exp \left[ -(0.2520+1-0.0625)\right] = 0.3044;\\ {\alpha _3}&= \exp ( - 0.2410 )=0.7858;\\ {\alpha _4}&=\exp ( - 0.2520)=0.7773;\\ {\alpha _5}&= \exp ( - 0.2359)=0.7899.\\ \end{aligned}$$

According to Eq. (16), the final weight $w_i, i = 1,2,3,4,5$ after normalization is shown as follows:

$$\begin{aligned} w _1&= 0.2340,\;\;\;\;\;w _2 = 0.0877,\;\;\;\;\;w_3 = 0.2265,\\ w_4&= 0.2241,\;\;\;\;\;w _5=0.2277. \end{aligned}$$

Obviously, we can obtain that there is a highly conflict between the evidence $m_2$ and other evidence. So $m_2$ is defined as an incredible evidence, and its weight is only 0.0877. Other evidence is supported by each other, so their weight are higher than $m_2$.

Finally, use the weight to modify the evidence, the results are:

$$\begin{aligned} m(A)=0.4872,\;\;\;m(B)=0.2078,\;\;\; m(C)=0.0790,\;\;\; m(A,C)=0.2260. \end{aligned}$$

After combine for 4 times by Dempster’s combination rule, the final results are given below:

$$\begin{aligned} m(A)=0.9837,\;\;\;m(B)=0.0021,\;\;\; m(C)=0.0110,\;\;\; m(A,C)=0.0032. \end{aligned}$$

The fusing results derived based on different combination rules are listed in Table 1. As illustrated in Table 1, when conflicting evidence is acquired, Dempster’s combination rule produces counter-intuitive results. When more BPAs are available, Murphy’s method (Murphy 2000), Deng et al.’s method (Deng et al. 2004) and the proposed method in this paper all provide reasonable results. However, Murphy’s method is only a simple arithmetic mean, without taking the relationship among the evidence into account. Deng et al.’s method uses the distance of evidence to determine the weight, which makes up the shortage of Murphy’s method, but the effect of evidence itself on weight is ignored. The experimental results show that the proposed method performs better than other methods. The reason is that the proposed method can better measure the uncertainty of evidence by Deng entropy and takes into consider the relationship between evidence and the evidence itself to define weight. Furthermore, it can strengthen the effect of credible evidence further and at the same time weaken the effect of incredible evidence further by the reward function and the penalty function.

**Table 1:** Evidence combination results based on different combination methods

Evidence	Method	*m*(*A*)	*m*(*B*)	*m*(*C*)	*m*(*AC*)	Target
$m_1,m_2$	Dempster’s method (Dempster 1967)	0	0.8969	0.1031	0	B
	Murphy’s method (Murphy 2000)	0.0964	0.8119	0.0917	0	B
	Deng et al.’s method (Deng et al. 2004)	0.0964	0.8119	0.0917	0	B
	The proposed method	0.0964	0.8119	0.0917	0	B
$m_1,m_2,m_3$	Dempster’s method (Dempster 1967)	0	0.6575	0.3425	0	B
	Murphy’s method (Murphy 2000)	0.4939	0.4180	0.0792	0.0090	A
	Deng et al.’s method (Deng et al. 2004)	0.4974	0.4054	0.0888	0.0084	A
	The proposed method	0.7614	0.1295	0.0961	0.0130	A
$m_1,m_2,m_3,m_4$	Dempster’s method (Dempster 1967)	0	0.3321	0.6679	0	C
	Murphy’s method (Murphy 2000)	0.8362	0.1147	0.0410	0.0081	A
	Deng et al.’s method (Deng et al. 2004)	0.9089	0.0444	0.0379	0.0089	A
	The proposed method	0.9379	0.0173	0.0361	0.0087	A
$m_1,m_2,m_3,m_4,m_5$	Dempster’s method (Dempster 1967)	0	0.1422	0.8578	0	C
	Murphy’s method (Murphy 2000)	0.9620	0.0210	0.0138	0.0032	A
	Deng et al.’s method (Deng et al. 2004)	0.9820	0.0039	0.0107	0.0034	A
	The proposed method	0.9837	0.0021	0.0110	0.0032	A

## Conclusion

Dempster’s combination rule will generate counter-intuitive results when dealing with highly conflicting evidence. In the past, more attention was paid to the relationship among the evidence in information fusion, and the research on the evidence itself is ignored. In the presented method, a novel belief entropy is taken as the uncertainty measure, and it is more accurate than the AM. Furthermore, the new method can efficiently handle conflicting evidence with better performance of convergence by jointly using the distance of evidence and the uncertainty measure.

In the future work, more factors will be analyzed and used in establishing the weight to construct more powerful evidence combination methods.
